# Modeling of Schottky diode and optimal matching circuit design for low power RF energy harvesting

**DOI:** 10.1016/j.heliyon.2024.e27792

**Published:** 2024-03-19

**Authors:** Abdelmalek Reddaf, Mounir Boudjerda, Islem Bouchachi, Badreddine Babes, Ali Elrashidi, Kareem M. AboRas, Enas Ali, Sherif S.M. Ghoneim, Mahmoud Elsisi

**Affiliations:** a Research Center in Industrial Technologies CRTI, ex CSC B. P. 64, Cheraga, Algiers, Algeria; b Ecole Nationale Supérieure des Technologies Avancées, Algiers, Algeria; c Engineering Mathematics Department, Faculty of Engineering, Alexandria University, Alexandria 21544, Egypt; d Electrical Engineering Department, University of Business and Technology, ArRawdah, Jeddah, 23435, Saudi Arabia; e Department of Electrical Power and Machines, Faculty of Engineering, Alexandria University, Alexandria 21544, Egypt; f Centre of Research Impact and Outcome, Chitkara University Institute of Engineering and Technology, Chitkara University, Rajpura-140401, Punjab, India; g Department of Electrical Engineering, College of Engineering, Taif University, P.O. BOX 11099, Taif 21944, Saudi Arabia; h Department of Electrical Engineering, National Kaohsiung University of Science and Technology, Kaohsiung 807618, Taiwan; i Department of Electrical Engineering, Faculty of Engineering at Shoubra, Benha University, Cairo 11629, Egypt

**Keywords:** Antenna, Energy harvesting, Matching network, Low power, Rectifier, Rectenna

## Abstract

This work designs and implements a single-stage rectifier-based RF energy harvesting device. This device integrates a receiving antenna and a rectifying circuit to convert ambient electromagnetic energy into useful DC power efficiently. The rectenna is carefully engineered with an optimal matching circuit, achieving high efficiency with a return loss of less than −10 dB. The design uses a practical model of the Schottky diode, where both RF and DC characteristics are derived through extensive experimental measurements. The results from both experiments and simulations confirm the effectiveness of the design, showing its proficiency in efficient RF energy harvesting under low-power conditions. The antenna produced operates in the wifi band with a gain close to 4 dBi and a bandwidth of 100 MHz. With a load resistance of 1600 Ω, the proposed device achieves an impressive RF-to-DC conversion efficiency of approximately 52% at a low incident power of −5 dBm. These findings highlight the potential and reliability of rectenna systems for practical and efficient RF energy harvesting applications. The study significantly contributes to our understanding of rectenna-based energy harvesting, providing valuable insights for future design considerations and applications in low-power RF systems.

## 1 Introduction

Harvesting energy from electromagnetic waves is emerging as a promising frontier in wireless power transfer. Electromagnetic waves, encompassing radio waves, microwaves, and various wireless communication signals [1], offer a valuable resource that can be harnessed and converted into useable electrical power. This capability opens up many applications, ranging from powering low-power wireless sensors, wearable devices, and IoT (Internet of Things) devices to providing wireless charging solutions for mobile devices and supplying energy to remote sensors in hard-to-reach locations. The foundation of electromagnetic wave energy harvesting lies in the principle of electromagnetic rectification [2]. Using specialized devices known as rectennas, short for rectifying antennas, we can capture the energy carried by these waves and convert it into electrical energy. This transformation process is pivotal for numerous technological advancements.

Energy harvesting from electromagnetic waves primarily involves two key stages [3]: energy capture and conversion. In the energy capture phase, an antenna or an array of antennas is employed to receive and accumulate electromagnetic waves. The design and characteristics of the antenna play a critical role in efficient energy capture, ensuring the extraction of a substantial energy yield [4]. Subsequently, the captured energy must be converted from the high-frequency alternating current (AC) form of electromagnetic waves into direct current (DC) electrical power, suitable for electronic devices or battery storage. This crucial conversion process is achieved through rectification, where the rectifier circuitry transforms the AC signal into useable DC current. One crucial aspect for RF energy harvesters is the choice of operating frequency. The 2.45 GHz industrial, scientific, and medical (ISM) band is attractive due to its balanced trade-off between signal attenuation and antenna size. The efficiency of energy harvesting from electromagnetic waves depends on several critical factors, including the power density of ambient electromagnetic waves, antenna design, rectification efficiency, and impedance matching between the antenna and the rectifier circuit.

Numerous approaches have been explored to enhance performance by utilizing broad [5] and multiple [6,7] frequency band antennas for harvesting power from diverse frequency ranges. However, despite delivering full power, both strategies are intricate, expensive, and time-consuming due to the necessity for a multi/wideband matching network. In contrast, comparable outcomes can be achieved by employing single-band antennas coupled with single-band matching networks [8], capable of operating at the same or different frequencies.

Nonetheless, the growing number of antennas and rectifier diodes makes the circuit expensive and impractical. Alternatively, techniques such as reducing diode threshold voltage [8], incorporating passive boosters [6], implementing multistage voltage multipliers [9], and utilizing an effective low-loss adaptation network have been proposed.

However, all the alternatives mentioned above boost output voltage at the cost of an increased manufacturing budget and circuit size. Considering these factors, our focus will be on optimizing matching network and diode parameters to maximize performance while maintaining circuit compactness.Achieving optimal matching and maximizing energy transfer efficiency are fundamental goals in successful energy harvesting endeavors [10]. In the design of our rectenna, our study unfolds in four distinct phases: Designing the antenna with specified parameters, including working frequency, gain, and input impedance. We employ a practical diode model to study the utilized diode, its equivalent circuit, and S-parameters. The electrical model incorporates only the intrinsic diode characteristics, with external parasitic elements assumed to be integrated into the diode's impedances. Diode parasites and nonlinear elements are extracted from S-parameters measurements at varying frequencies, obviating the need for separate curve measurements (*I–V* or *C–V*). This research facilitates diode modeling using a single measurement configuration, leveraging a vector network analyzer.After the design of the matching networks on the both sides of the diode. Finally, it involves an optimization procedure that integrates the preceding phases and ensures achieving the desired efficiency and input return loss. The novelty and technical contributions of the article include.

1. A conceptual development of a new optimized rectenna;
2. Creation of a compact antenna (47 × 35 mm) with good performance in terms of operational bandwidth (2.45 GHz) and high gain (6 dBi);
3. Comparison of the proposed rectenna with the previously reported rectennas in literature in the form of a table to show the uniqueness of this work.

## 2 Design of the rectifier circuit

An RF energy harvester's primary goal is to efficiently convert electromagnetic energy from nearby RF sources into a stable direct current (DC) electricity supply. A typical rectenna (rectifying antenna) configuration comprises several essential components, which include a receiving antenna, an impedance-matching network, a rectifier, a DC filter, and a load [[6], [7], [8], [9], [10]]. For the streamlined design of our circuit, we have chosen a series topology. The first component in our design is a Wi-Fi RF band-compatible antenna, meticulously selected to maximize power transmission to the rectifier circuit. Achieving optimal power transfer requires the inclusion of a matching circuit.

**Fig. 1:**
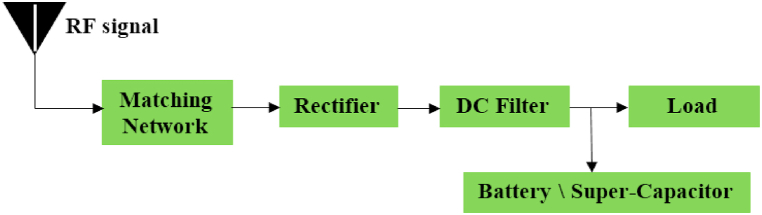
General block diagram of rectenna.

Our rectifier circuit is composed of a series-connected low-pass filter and a Schottky diode, as depicted in Fig. 1. In this work, we adhere to specific design considerations to enhance overall efficiency. These guiding principles include [11]: type of antenna, type of rectifier, diodes, filtering capacitors, load considerations, and impedance matching.

## 3 Pratic model of Schottky diode

Schottky diodes are preferred for energy harvesting applications due to their unique characteristics and associated benefits [12]. Here are several reasons why Schottky diodes are well-suited for energy harvesting.

### 3.1 Low forward voltage drop

Schottky diodes exhibit a lower forward voltage drop when compared to standard p-n junction diodes [13]. This characteristic allows them to efficiently rectify lower input voltages and minimize power losses during the energy conversion. This feature makes Schottky diodes ideal for efficient energy harvesting, particularly when dealing with low-power sources.

### 3.2 Fast switching speed

Schottky diodes have a rapid switching speed, which means they can swiftly respond to changes in the input signal [14]. This quality is highly advantageous in energy harvesting systems, especially when dealing with rapidly varying or intermittent energy sources. The diodes can efficiently capture and convert energy from rapidly changing RF signals.

### 3.3 Compact size and low profile

Schottky diodes are available in small package sizes and have a low profile [15]. This compact form factor makes them well-suited for integration into miniaturized energy harvesting systems, where space constraints are often a concern. Their compact size allows for efficient and discreet placement within devices and systems.

**Fig. 2:**
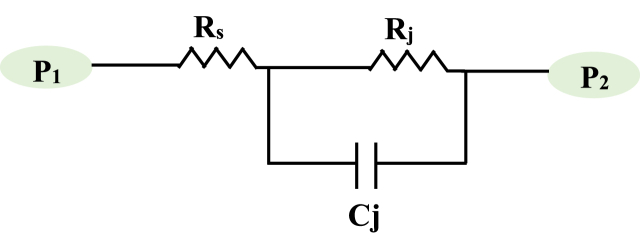
The equivalent electrical circuit of a Schottky diode.

Fig. 2 depicts the equivalent circuit of a Schottky diode, with *C*_*j*_ representing the junction capacitance in Farad, *R*_*s*_ representing the series resistance in *Ω* (this resistance is caused by the inability of charges to move through the crystal lattice structure easily, and it models the diode's losses by joule effect), and *R*_*j*_ representing the junction resistance in *Ω*. The variable junction resistor models that the diode is either conducting or blocked. There are two kinds of Schottky diodes. The first type of silicon is n-type silicon, which has a high barrier and low *R*_*s*_ values. The second type of silicon is p-type, which has a low barrier and high *R*_*s*_. The p-type Schottky diode is suggested for low input power level applications because it has a larger output voltage than the n-type [13]. HSMS-285X (n-type) and HSMS-286X (p-type) are the most often used Schottky diodes [15]. To simulate the real behavior of the Schottky diode in our experimental circuit, we have created a practical diode models, consisting of two key components.

### 3.4 Modeling DC behavior of the Schottky diode

To model the DC behavior of the Schottky diode accurately, we initiated the process with a standard diode and employed an optimization method. This approach involved determining key diode model parameters, which include the saturation Current *I*_*s*_, the series resistance *R*_*s*,_ and the ideality factor *N*.

**Fig. 3:**
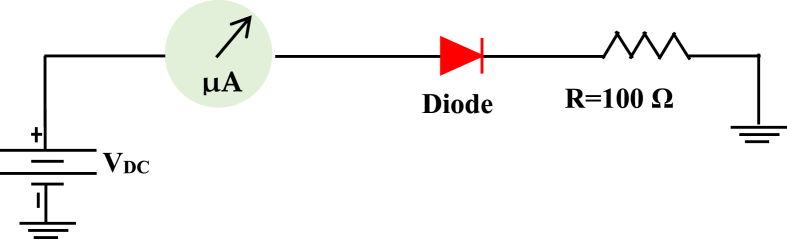
Agilent ADS model for Simulated I–V characteristics.

These parameters were determined through a meticulous optimization process, incorporating the superposition of experimental curve *(I–V*) characterization data of the real diode; whose (*I–V*) assembly is presented in Fig. 3.

### 3.5 Modeling RF behavior of the Schottky diode

**Fig. 4:**
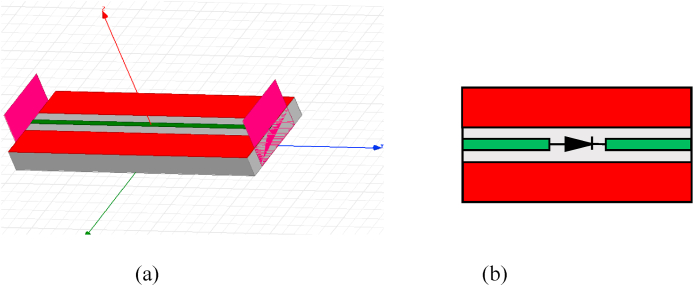
The microwave test for the schottky diode: a) without diode, b) with diode.

**Fig. 5:**
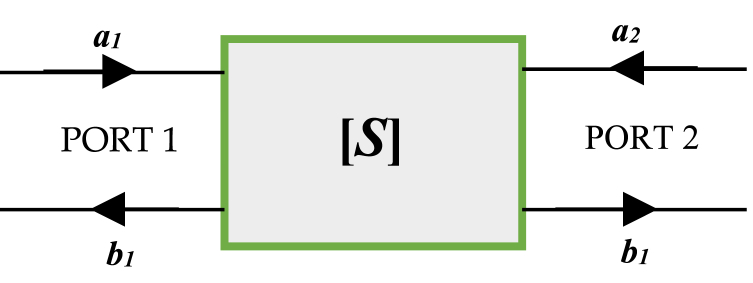
Definition of the two-port S-parameter network.

Fig. 4 (a,b) represents the microwave test (measurement device) for the schottky diode. Once the diode model for the Schottky diode is established, a practical approach for determining matching networks at the operating frequency of 2.45 GHz is to employ *S-*parameters. Fig. 5 illustrates the decomposition of the matrix [*S*] as it relates to the inputs and outputs of the quadruple. Eq. (1) is used to translate the relationship between the components of the matrix [*S*]:

(1) $$\left[\begin{array}{c} b_{1}\\ a_{1} \end{array}\right]=\left[\begin{array}{cc} S_{11} & S_{12}\\ S_{21} & S_{22} \end{array}\right]\left[\begin{array}{c} b_{2}\\ a_{2} \end{array}\right]$$

**Fig. 6:**
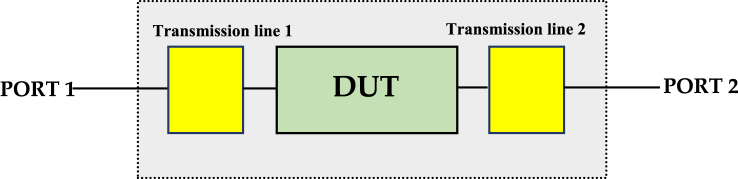
Test fixture configuration showing the measurement and device planes.

**Fig. 7:**
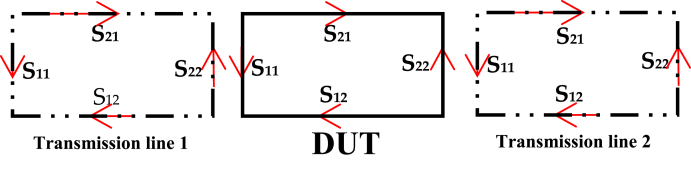
Signal flow diagram of the S parameters of the test device combined with the forward two-port transmission lines.

**Fig. 8:**
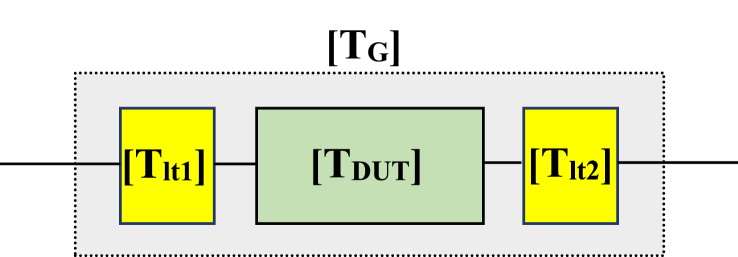
Cascade [T] matrix model.

By replacing the diode with its corresponding matrix [*S*], it becomes feasible to analyze the behavior of the diode model over a wide frequency range. This subsection provides a concise and structured explanation of how the diffusion matrix [*S*] is applied to determine matching networks for the diode model, making it easier for the reader to follow the process. In modeling the Schottky diode, a crucial step involves using a de-embedding technique to extract S-parameters for the device under test (DUT). This technique is commonly utilized in radio frequency and signal integrity measurements, allowing for precise analysis and modeling. Fig. 6 illustrates the design composition, which consists of three serial components: transmission line 1, the DUT (Schottky diode model), and transmission line 2. The de-embedding process relies on the measured data of a global set and is based on known S-parameters of the transmission line. Each component in the system has its own matrix S, contributing to the accuracy of the analysis as depicted in Fig. 7. The primary goal of this de-embedding technique is to eliminate the impact of the transmission line in which the DUT is inserted. By removing the influence of the transmission line, the analysis can focus solely on the behavior of the DUT, enhancing modeling precision. After measuring the S-parameters, the de-embedding process is carried out using a multi-step approach, which involves the bidirectional transformation of the S-parameters into corresponding transfer diffusion parameters (*T*). This process is fundamental for accurate modeling and analysis. Fig. 8 illustrates the transition from (*S*) parameters to transfer diffusion parameters (*T*), a critical step in de-embedding. Eq. (2) translates the relationship between the matrix components [*T*]. Eq. (3) presents the general matrix of the overall setup, providing a comprehensive representation of the system. This approach is well-established for two- and four-port networks and has recently been extended to the multiport case. It offers an alternative analytical formulation for offloading a multiport device, even when only forward and downstream transmission line parameters (*S*) are known.The process automatically deduces their transfer matrix (*T*) by applying Eqs. (3), (4), (5), (6) and replacing the measurement results after deducing the *S* parameters of the device under test.

(2) $$\left[\begin{array}{c} b_{1}\\ a_{1} \end{array}\right]=\left[\begin{array}{cc} T_{11} & T_{12}\\ T_{21} & T_{22} \end{array}\right]\left[\begin{array}{c} a_{2}\\ b_{2} \end{array}\right]$$

(3) $$\left[T_{DUT}\right]={\left[T_{lt1}\right]}^{-1}{\left[T_{G}\right]}^{1}{\left[T_{lt2}\right]}^{-1}$$

(4) $$\left[T_{G}\right]={\left[T_{lt1}\right]}^{1}{\left[T_{DUT}\right]}^{1}{\left[T_{lt2}\right]}^{1}$$

(5) $$\left[\begin{array}{cc} S_{11} & S_{12}\\ S_{21} & S_{22} \end{array}\right]=\left[\begin{array}{cc} \frac{T_{12}}{T_{22}} & \frac{T_{11}T_{22}-T_{12}T_{21}}{T_{22}}\\ \frac{1}{T_{22}} & -\frac{T_{21}}{T_{22}} \end{array}\right]$$

(6) $$\left[\begin{array}{cc} T_{11} & T_{12}\\ T_{21} & T_{22} \end{array}\right]=\left[\begin{array}{cc} -\frac{S_{11}S_{22}-S_{12}S_{21}}{S_{21}} & \frac{S_{11}}{S_{21}}\\ -\frac{S_{22}}{S_{21}} & \frac{1}{S_{21}} \end{array}\right]$$

## 4 Matching circuit

**Fig. 9:**
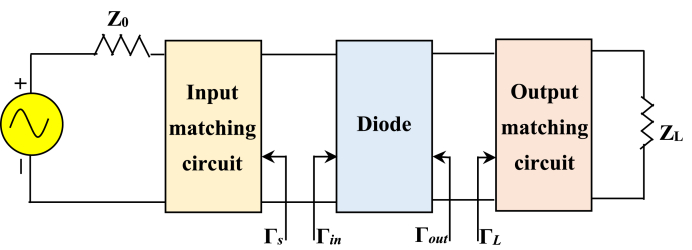
A lossless matching network before and after diode.

Efficient matching is a critical component in enhancing energy harvesting systems' overall performance and efficiency. However, the matching circuit faces several challenges in practice: The nonlinear behavior of diodes, including Schottky diodes, can impact the efficiency of the energy harvesting system; and the low amplitude (Voltage) of the input signal can limit the system's performance [16]. The impedance of the rectifier varies with factors such as input power, frequency, and load resistance. This variability can restrict circuit performance to a narrow frequency band and limit the maximum power transfer [17]. So, the matching networks play a pivotal role in addressing the challenges and enhancing system efficiency. They can be realized using either lumped elements (Resistor, Inductor, and Capacitor) or distributed elements (Microstrip lines & Stubs). Effective matching networks seek to be lossless, minimizing power dissipation and ensuring that the impedance observed within the network is *Z*_*0*_, the transmission line's characteristic impedance [18]. This design principle reduces reflection waves on the transmission line [19]. A general matching network requires at least two degrees of freedom, provided by the values of the two reactive components in the matching circuit. Utilizing tools like the Smith chart enables rapid and precise design of matching networks. In the design of the rectenna, the primary objective is to maximize power transmission to the load. This goal aligns closely with the principles used in designing maximum gain amplifiers. Achieving maximum power transfer involves employing two conjugate matching networks, a configuration similar to that used in high-gain amplifiers, as illustrated in Fig. 9. For that to increase the amount of power given to the load, we can determine the corresponding load impedance *Z* by performing an impedance transformation along the line, considering the known input impedance *Z*_*in*_ [20] which presents in Eq. (7). To maximize the power (*P*), we discriminate between the real and imaginary components. This helps us to determine the best load impedance for maximum power transmission to the load.

(7) $$R_{in}=R_{g},X_{in}={-X}_{g}$$

Where *R*_*g*_ and *X*_*g*_ are the real and imaginary components of RF generator impedance. Eq. (8) determines this condition, which is known as conjugate matching, and it results in maximum power transfer to the load for fixed generator impedance [20]:

(8) $$Z_{in}=Z_{g}^{*}$$

where *V*_*g*_ is the generator voltage, the maximum delivered power (*P*) can be determined by Eq. (9):

(9) $$P=\frac{1}{2}{\left|V_{g}\right|}^{2}\frac{1}{4R_{g}}$$

To attain optimal power transfer, the reflection coefficients of the diode's input (*Γ*_*in*_) and output (*Γ*_*out*_) must be equal to the conjugate of the reflection coefficients observed when looking into the source (*Γ*_*s*_) and the load *(Γ*_*L*_), as expressed in Eqs. (10), (11)):

(10) $$\Gamma_{in}=\Gamma_{s}^{*}$$

(11) $$\Gamma_{out}=\Gamma_{L}^{*}$$

We must also consider that the output matching network plays the role of the DC pass filter. To recap, we have seen two types of matching to consider: impedance matching, which aims to eliminate reflection, and conjugate matching, which seeks to maximize energy transfer.

Striking the right balance between these two matching techniques is crucial to achieve optimal efficacy while minimizing return loss to a level of −10 dB or below. This delicate compromise ensures efficient energy capture and conversion, leading to a highly effective and reliable rectenna system.

## 5 Rectenna efficiency

Rectenna circuits are often distinguished by two efficiencies: RF-DC conversion efficiency and total efficiency [21]. The first efficiency describes the rectifier's ability to supply continuous electrical power to the load from the RF energy supplied by the receiving system or any other microwave energy source. This efficiency is the primary goal of a conversion circuit optimization method. This last one relates to the ratio of *P*_*DC*_, the power recovered at the rectifier's output, to *P*_*RF*_, the power injected at the rectifier's input using a microwave source. It is calculated using the following Eq. (12):

(12) $$\eta \%=100*\frac{P_{DC}}{P_{RF}}$$

However, due to the non-linear nature of the conversion process, the efficiency is best within a small range surrounding the point where the optimization occurs [22]. Indeed, the Schottky diode's efficiency and impedance depend on the voltage applied to its terminals. This impedance's change causes a mismatch that negatively impacts conversion efficiency. The second efficiency is the difference between the RF input power *P*_*RF*_ and the DC output power *P*_*DC*_, which is obtained from the load's *R*_*L*_ terminals. Where as shown below, the *P*_*DC*_ power is determined by Eq. (13):

(13) $$P_{DC}=\frac{V_{DC}^{2}}{R_{L}}$$

The reverse breakdown voltage *V*_*br*_ and load resistance *R*_*L*_ limit the maximum DC voltage across the diode, *V*_*DC*_ as shown in Eq. (14):

(14) $$V_{DC}=\frac{V_{br}}{2}$$

The literature contains numerous definitions of input power [23]. The maximum power that the source or the receiving antenna can send to a 50 load is referred to as the RF power, and in this instance, reflection losses are taken into account [21]. It can be thought of as the RF power that is effectively communicated to the diode, excluding losses due to reflection [24]. The last definition, which is broader, discusses how well the entire antenna circuit can transform the RF energy it receives into DC energy. Frequently, the Friis equation is used to estimate the amount of power that the antenna is anticipated to receive [25], which is calculated by formula (15):

(15) $$P_{RF}=P_{e}G_{e}G_{r}{\left(\frac{\lambda }{4\pi r}\right)}^{2}$$

It gives the received RF power as a function of the sent power *P*_*e*_, the maximum gains of the sending and receiving antennas, and the losses in free space, which depend on the frequency and the distance *r* between the two antennas.

## 6 Results and discussion

**Fig. 10:**
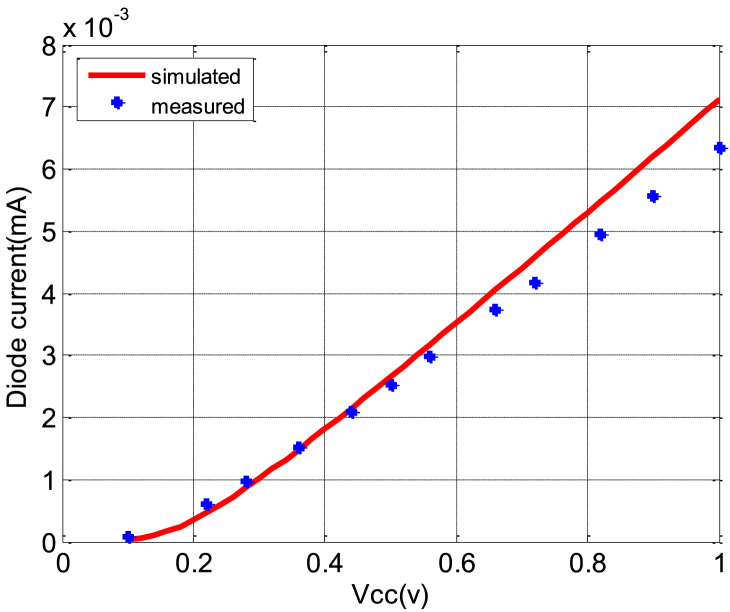
Simulated and measured (I–V) characteristics.

As previously noted, the DC model is formulated by fitting the (*I–V*) characteristics of the Schottky diode HSMS 2850, as illustrated in Fig. 10. Through an optimization process, we derived the diode's parameters, aligning the simulated (*I–V*) characteristics with the experimental data. Table 1 presents the optimized parameter values, closely resembling the real diode's characteristics.

**Table 1:** Optimized parameter values of DC model of Schottky diode.

Parameters	Value
*I*_*s*_	1.9E-8
*R*_*s*_	5.02 Ω
*N*	1.02

**Fig. 11:**
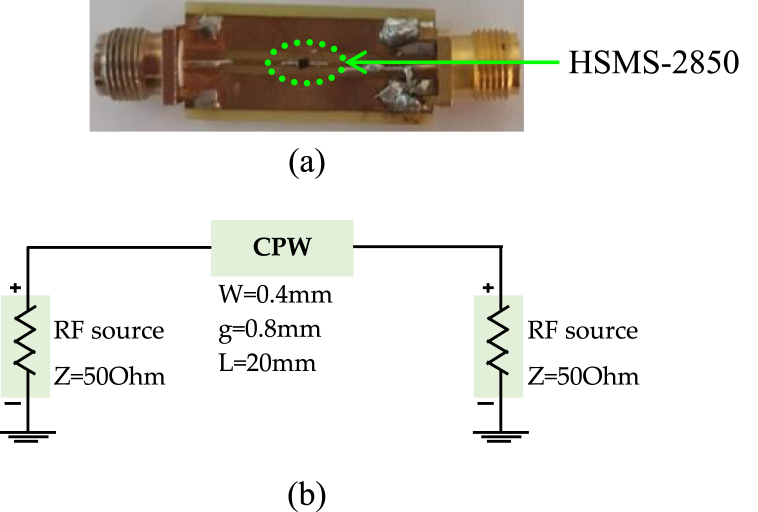
The coplanar line utilized for RF characterization; a) experimental: b) Agilent ADS model of coplanar line without Schottky diode.

**Fig. 12:**
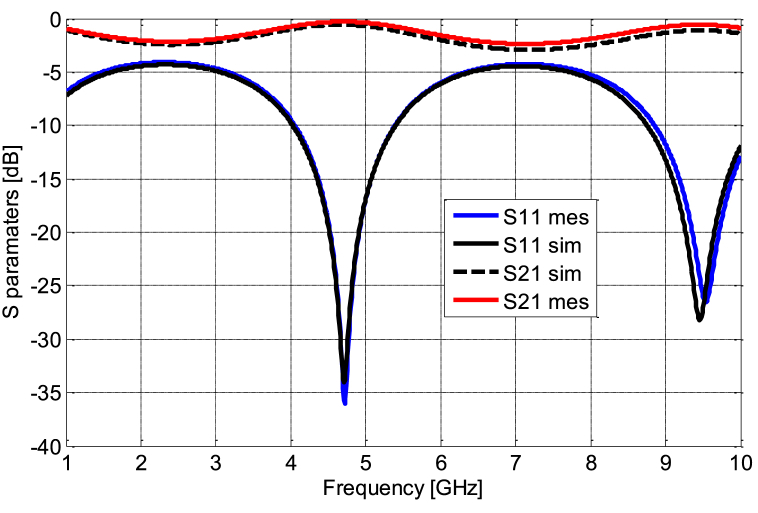
Comparison of *S*_*11*_ and *S*_*21*_ for the measured and modeled coplanar line without Schottky diode.

**Fig. 13:**
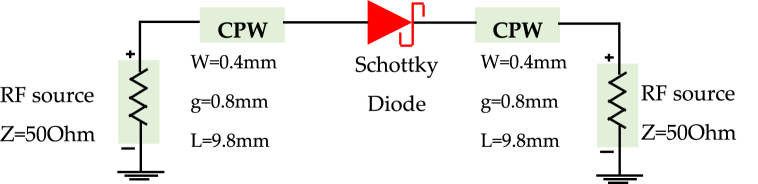
Model of coplanar line with Schottky diode.

**Fig. 14:**
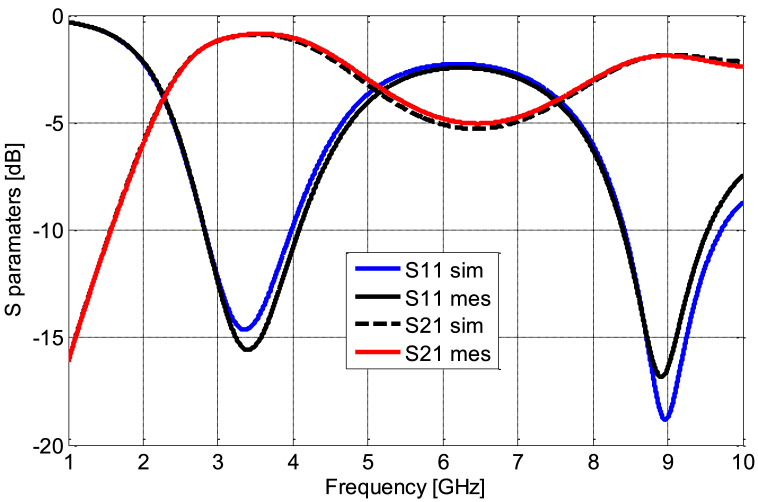
Comparison of *S*_*11*_ and *S*_*21*_ for the measured and modeled coplanar line Schottky diode.

**Fig. 15:**
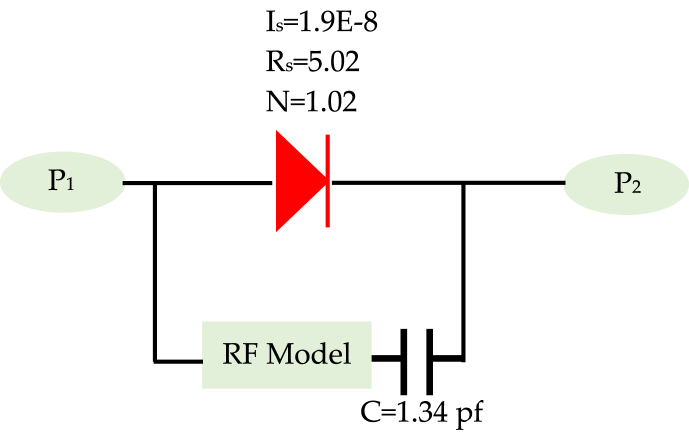
Agilent ADS model of Schottky diode HSMS-2850.

Fig. 11 (a, b) presents the coplanar line utilized for diode characterization, with the RF model of the simple coplanar line, excluding the diode, established based on its dimensional attributes using Advanced Design System (ADS) software. Meanwhile, Fig. 12 highlights the similarity between *S*_*11*_ and *S*_*21*_ parameters between the measurements and the model of the line. We followed the de-embedding procedure outlined in the previous section to assess the integrated diode's impact in the middle of the line and acquire S-parameters for the complete device. By employing these steps, we obtained the actual S-parameters closely resembling those of the Schottky HSMS 2850 diode, as depicted in Fig. 13. The comparison between the measured and simulated S-parameters for the coplanar structure with the diode is illustrated in Fig. 14, revealing a strong agreement between simulation and measurement. Subsequently, building upon the DC and RF models, we formulated an authentic representation of the diode, a critical component within our rectenna system presented in Fig. 15.

**Fig. 16:**
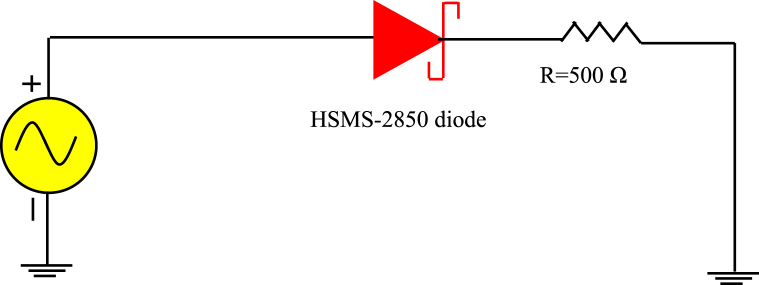
Circuit for the AC characteristics.

**Fig. 17:**
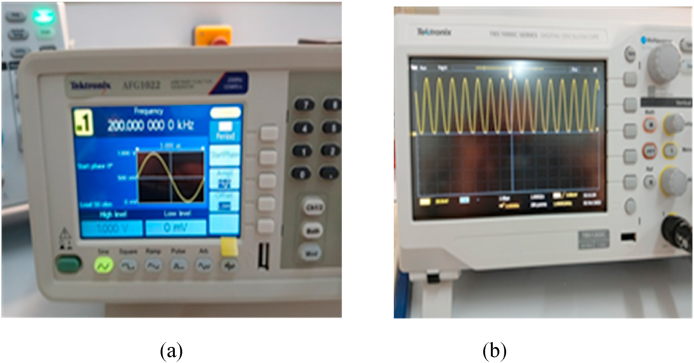
The equipment used in AC measurement of the real diode: (a) Tektronix AFG 1022 signal generator, (b) Tektronix TBS1102C Digital oscilloscope.

**Fig. 18:**
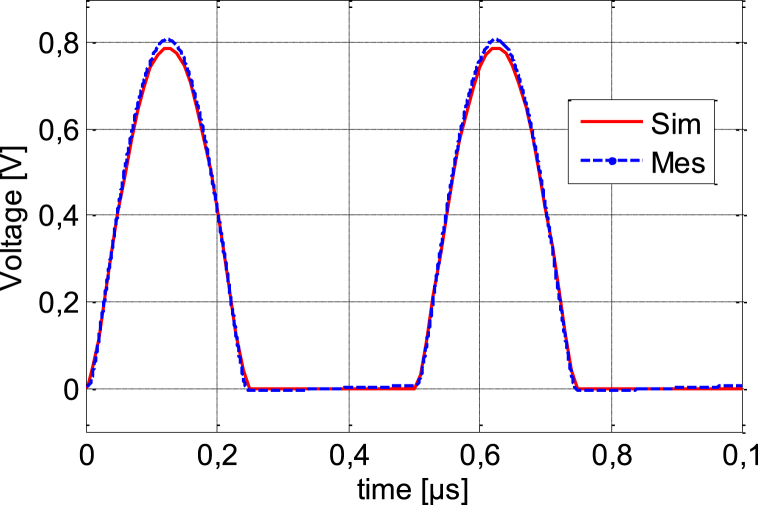
Comparison of the simulated and measured terminal voltages of the model and real an HSMS-2850 Schottky diode at 200 KHz.

**Fig. 19:**
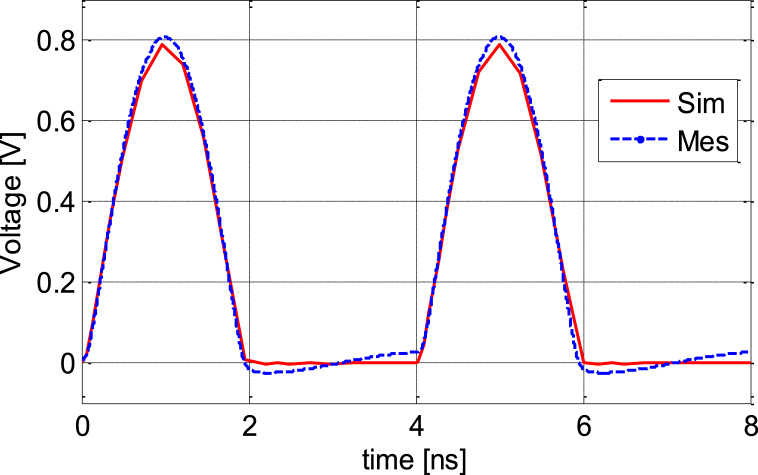
Comparison of the simulated and measured terminal voltages of model and real an HSMS-2850 Schottky diode at 100 MHz.

A low frequency circuit with an HSMS-2850 diode and an AC source with voltage amplitude *U* = 1V with resistance *R* = 500W is shown in Fig. 16. Fig. 17 shows The Tektronix AFG 1022 signal generator and the Tektronix TBS1102C Digital oscilloscope; which are used to compare the output of the measurement with the simulation of the AC characteristic of the device at the frequency *f* = 200 KHz and 100 MHz. The comparison of the observed and simulated voltages across the HSMS-2850 diode is shown in Fig. 18, Fig. 19. The simulated result and measured result correspond rather well.

A rectangular patch antenna is design heavily depends on two key factors: the resonance frequency and the choice of substrate material [26]. Typically, designing such an antenna involves two main stages: (i) calculating the patch dimensions: in this step, the patch dimensions are determined. These dimensions are essential for achieving resonance at the desired frequency; (*ii*) Determining the transition line and feeder dimensions: to achieve the desired input impedance, which is often set to 50 Ω, the dimensions of the transition line and the feeder need to be carefully calculated.

**Fig. 20:**
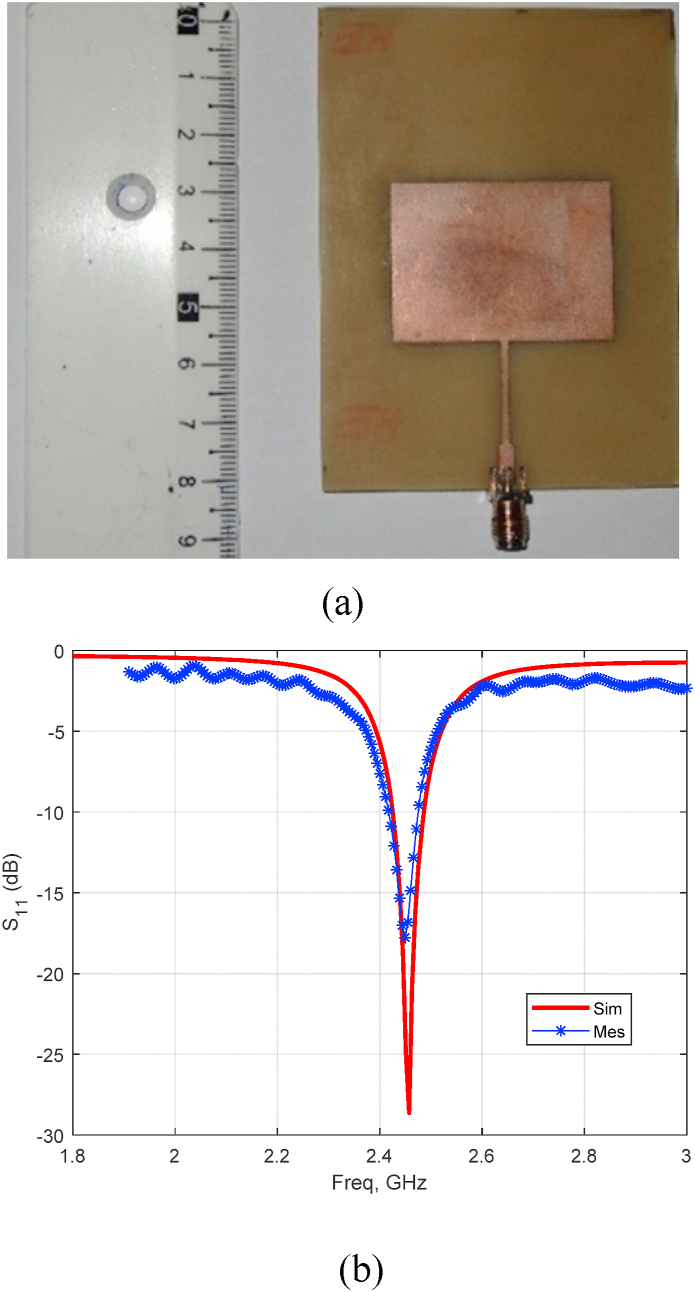
Photograph of the fabricated antenna (a), reflection coefficient *S*_*11*_ (b).

For our specific design, we utilized an FR4 epoxy substrate with a permittivity (*εᵣ*) 4.4, a thickness (h) of 1.57 mm, and a loss tangent 0.02. Fig. 20 (a) and (b) depict the antenna itself and its reflection coefficient *S*_*11*_, respectively, visually representing the design and its performance. The experimental results show a good agreement with simulation results as depicted. Also, the results demonstrate that the simulated and the manufactured antenna have the same bandwidth and resonance frequency (*fr*measured = 2.449 GHz and *fr* simulated = 2.457 GHz).

Optimizing matching in an energy harvesting system is a multi-faceted process that involves careful consideration of several factors, including the energy source's characteristics, the harvesting device's properties, and the impedance matching techniques employed. By methodically designing and fine-tuning various parameters within the energy harvesting system, such as resonant frequencies, impedance matching networks, and load conditions, it has previously mentioned, when it comes to matching the antenna with the rectifier circuit, the scattering matrix of the diode plays a crucial role. This matrix enables the calculation of reflection coefficients at the rectifier's input and output. In our case, this matrix has been computed using Advanced Design System (ADS) and is provided for a frequency of 2.45 GHz:

(16) $$\left[S\right]=\left[\begin{array}{cc} -1.026 & -8.191\\ -8.191 & -1.026 \end{array}\right]$$

**Fig. 21:**
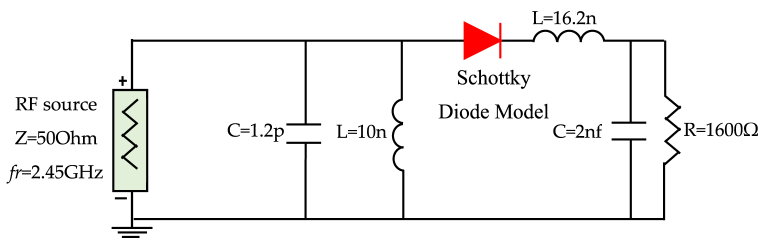
Diagram of rectenna circuit according to the design.

**Fig. 22:**
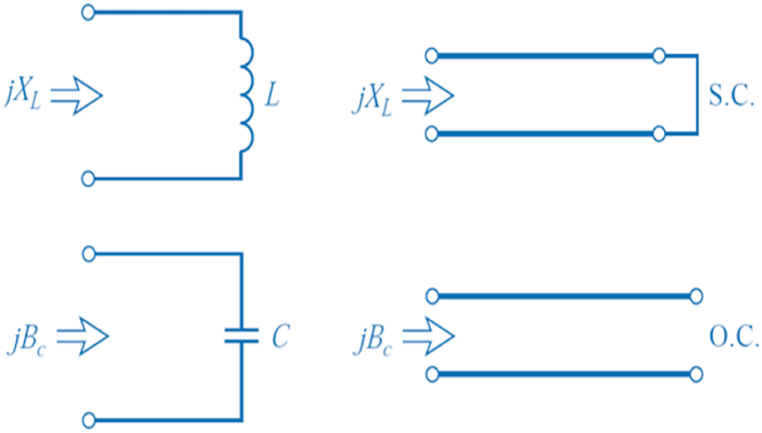
Lumped elements to distributed elements equivalences.

To match the components, we have incorporated a capacitor (*C*_*3*_) and inductance (*L*_*1*_) in parallel, forming an *LC* resonator between the antenna and diode. This network also functions as a band-pass filter, effectively eliminating harmonic waves. The matching network between the diode and the load also comprises a capacitor (*C*_*4*_) and an inductance (*L*_*6*_). The values of *C*_*3*_, *L*_*1*_, *C*_*4*_, and *L*_*6*_ were determined through an optimization process to maximize efficiency while maintaining a return loss of −10 dB. The final configuration of the rectenna is presented in Fig. 21. This particular topology offers the advantage of transitioning from lumped elements to distributed elements, which is realized through the conversion method depicted in Fig. 22. This approach proves highly beneficial for optimizing the energy harvesting system.

**Fig. 23:**
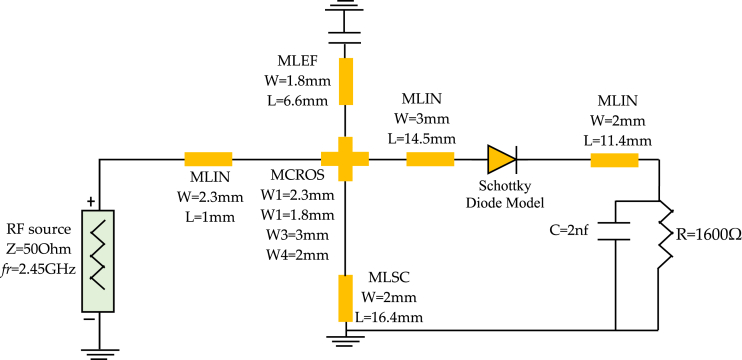
Diagram of rectenna circuit including microstrip components.

To determine the dimensions of the stubs and transmission line segment utilized to replace capacitance *C*_*3*_ and inductances *L*_*1*_ and *L*_*6*_, we employed a programming interface that connects MATLAB with HFSS (High-Frequency Structure Simulator). Concerning _C4_, we chose a capacitor for its role in smoothing the rectified signal and isolating the load from the rectifier, ensuring it has no significant impact on the matching. The final configuration is depicted in Fig. 23. In the literature, it is often mentioned that maximum efficiency is achieved when the power captured by the antenna equals or exceeds 10 dBm. However, in practical scenarios, this criterion usually applies only to very powerful energy sources close to the receiving antenna. Realistically, captured energy levels tend to hover around −10 dBm. It led us to design our device to achieve maximum efficiency around −5 dBm.

**Fig. 24:**
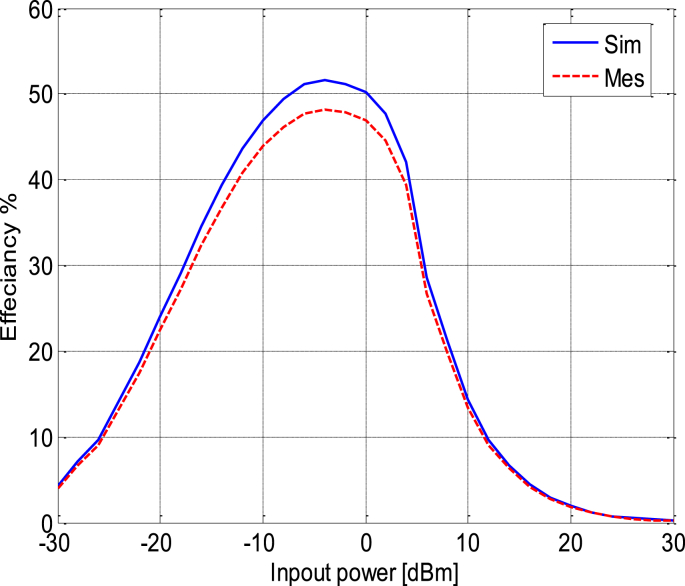
RF-to-DC conversion efficiency of the simulated and measured rectenna with the input power.

**Fig. 25:**
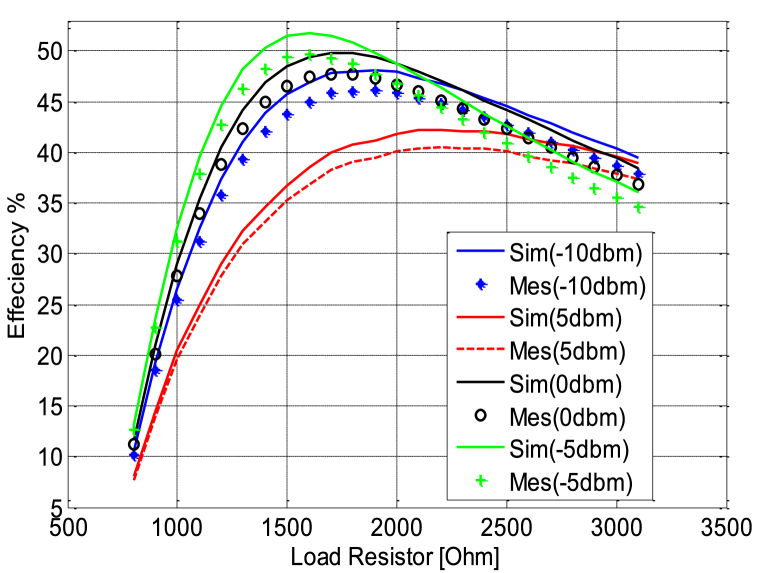
RF-to-DC conversion efficiency of the simulated and measured rectenna with the load resistor.

**Fig. 26:**
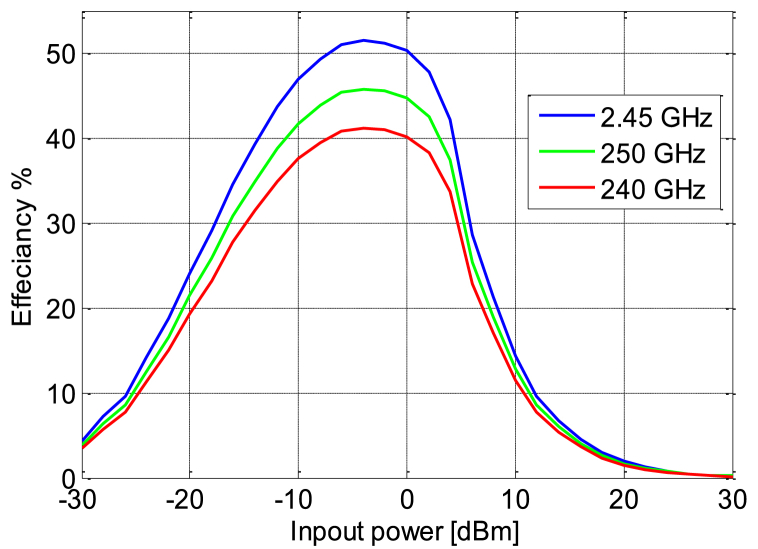
RF-to-DC conversion efficiency of rectenna with input power as a function of frequency.

Fig. 24 shows the variation of simulated and measured circuit efficiency fluctuates as the circuit input power increases from −30 to 30 dBm.To evaluate the DC power output characteristics while varying the load resistance (*R*) connected in parallel to capacitor *C*_*4*_, we conducted experiments. We systematically swept the load resistance parameter over a range from 500 Ω to 3.5 KΩ with input power varying from −10 to 5 dBm. Fig. 25 shows the variation in efficiency as a function of load by varying the power of input, the harvested DC power reaches its peak when the load resistance is set to 1600 Ω. This observation underscores the importance of choosing the optimal load resistance value for maximizing the harvested DC power within the given configuration; this figure shows that the circuit designed based on the low power approximately −5 dBm whose efficiency is higher compared to other powers. Meanwhile Fig. 26 illustrates the variation in efficiency as a function of the operating frequency of the patch antenna; therefore the circuit is designed to work at full power at the frequency 2.45 GHz, which is shown in Fig. 26 on the other hand at the two frequencies presented antenna bandwidth is less efficient.

**Fig. 27:**
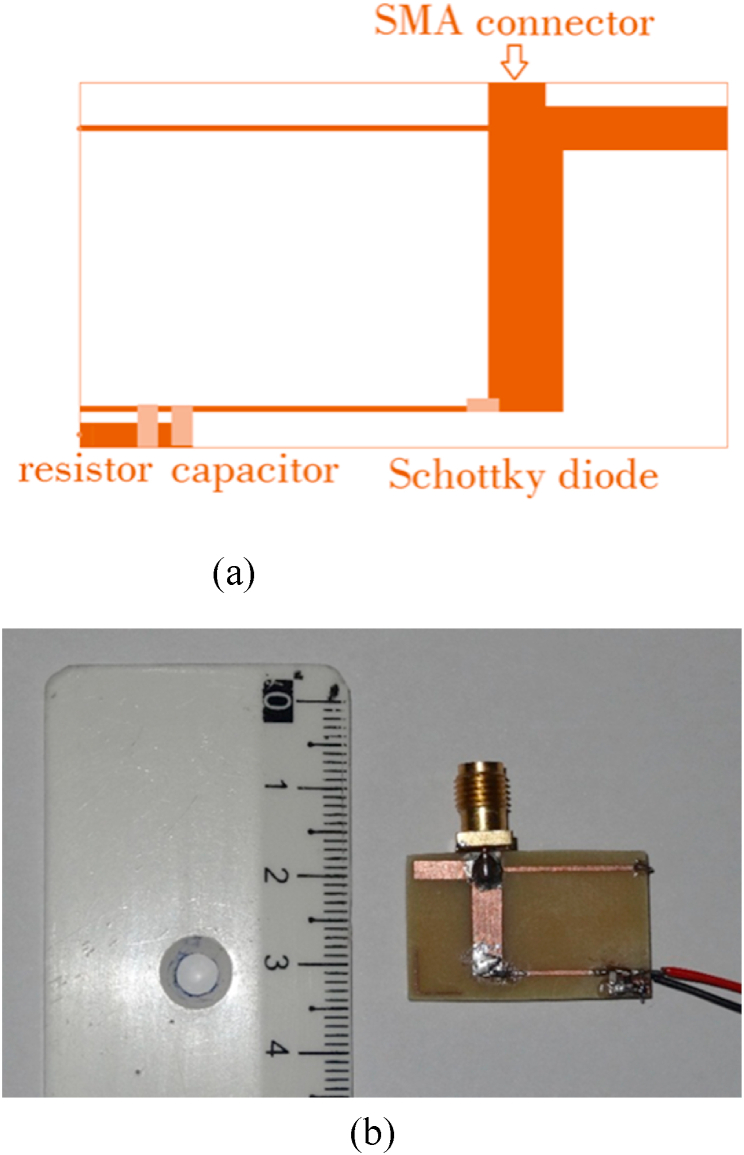
The prototype of the proposed rectiﬁer: (a) layout, (b) fabricated.

**Fig. 28:**
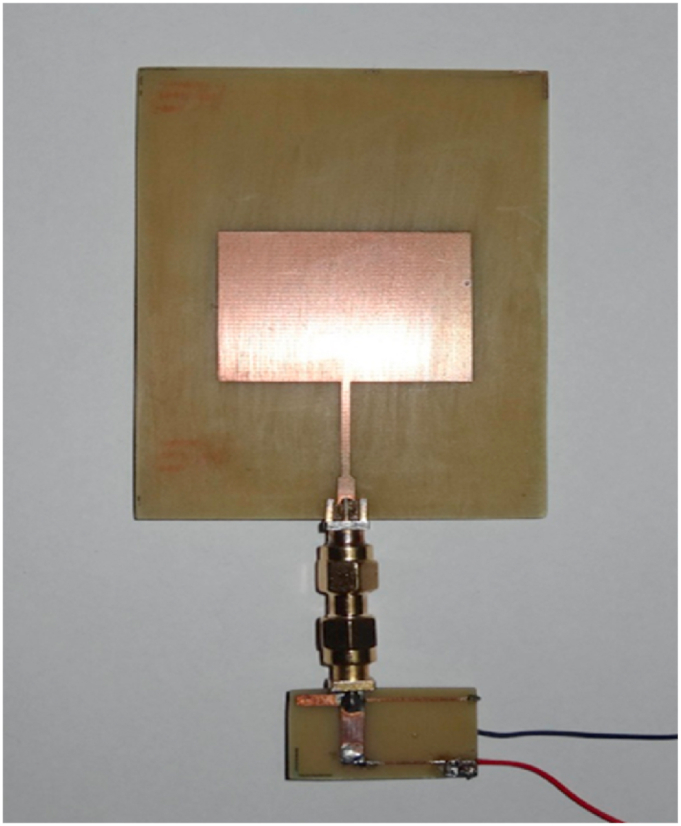
Photograph of the realized rectenna.

The rectifier circuit was constructed on the same substrate used for the antenna, which is FR4 epoxy; with dimensions measuring 17 mm × 27 mm. Fig. 27 (a, b) provides both a layout and a photograph of the rectifier, offering a visual representation of the circuit.To complete the rectenna system, the receiving antenna and the rectifying circuit were interconnected via an SMA connector with a characteristic impedance of 50 Ω, as depicted in Fig. 28. This connector forms the crucial link between the antenna and the rectifier, ensuring the efficient transfer of the captured RF energy for rectification and subsequent utilization.

**Fig. 29:**
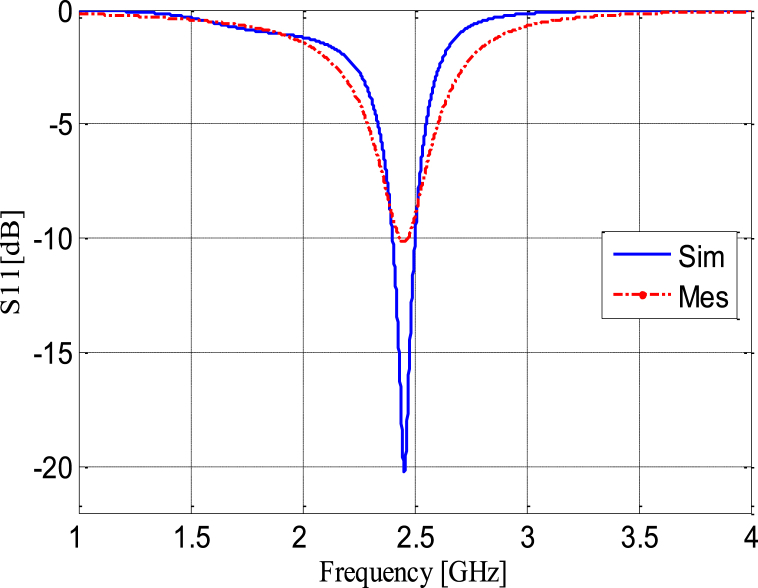
Simulated and measured reflection coefficient of the rectifier as a function of frequency.

**Fig. 30:**
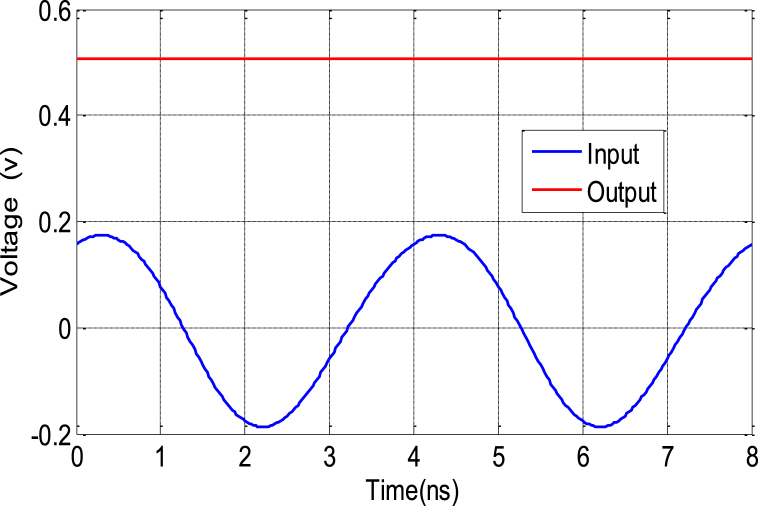
Simulated input and output voltage.

The reflection coefficient of the rectifier is visualized in Fig. 29, providing insight into the performance of the rectifying circuit when presented with incoming RF signals. Meanwhile, Fig. 30 illustrates the temporal evolution of both the input and output voltages of the RF-DC converter operating at a frequency of 2.45 GHz. In Fig. 30, it is evident that the input voltage exhibits a sinusoidal pattern, which is characteristic of RF signals. In contrast, the output voltage remains remarkably constant over time. The maximum input voltage reaches +200 mV, while the rectified output voltage stabilizes at around 500 mV. This behavior highlights the rectifier's ability to efficiently convert varying RF signals into a relatively stable DC voltage, a crucial step in the energy harvesting process.

**Fig. 31:**
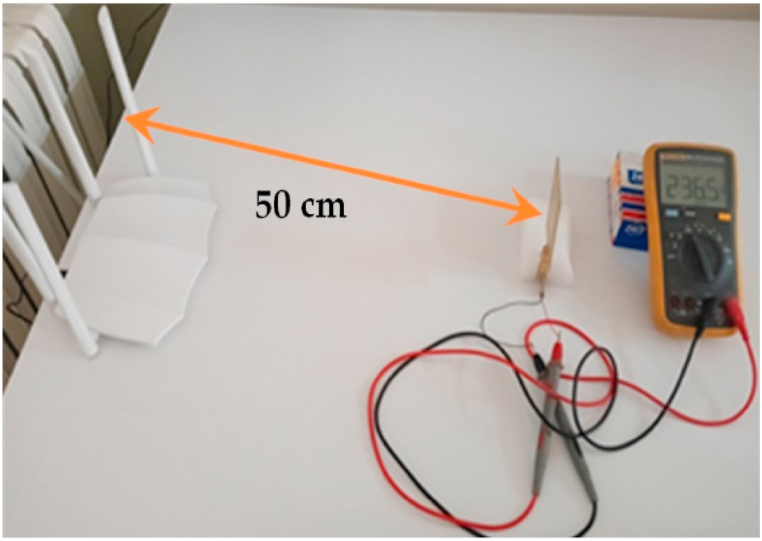
Measuring rectenna in a typical indoor environment with a voltmeter.

**Fig. 32:**
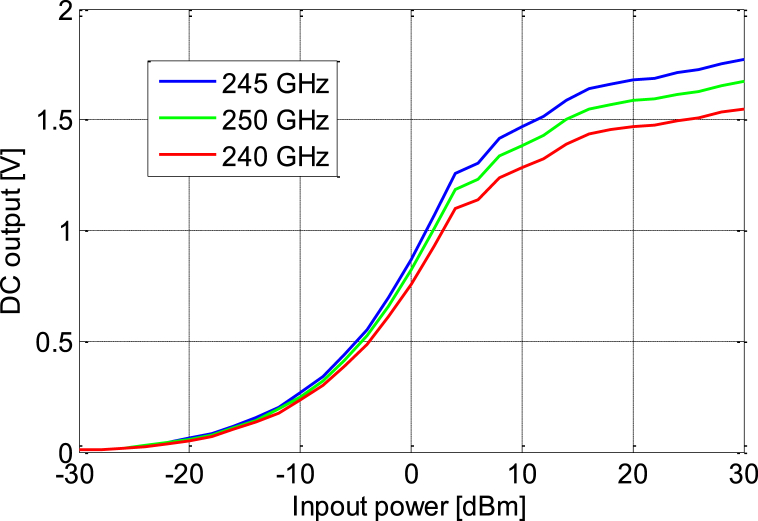
Output voltage versus frequency.

Fig. 31 presents a real example of an RF source 50 cm away for our rectenna with a power of 60 mW; then rectenna provides a voltage of approximately 236 mv, following the FRIIS theorem whose gains are between 3.5 and 4 dBi, so the RF source is at −10 dBm, consequently the converter provides a voltage of around 280 mv.The effect of matching circuit on the rectifier output is very important so the voltage is higher for the frequency 2.45 GHz compared to the other frequencies, since the circuit is based on the frequency approximately 2.45 GHz, so Fig. 32 actually shows this.

Table 2 provides a comprehensive comparison of performance metrics between the proposed rectifier circuit and other relevant circuit designs. The results clearly demonstrate that the proposed rectifier circuit outperforms all other designs in terms of conversion efficiency while operating with the lowest input power and smaller physical size. if by comparing the size and filling of other designs our circuit is better classified and even in terms of cost too.This highlights the significant progress and superior performance achieved in the proposed work, making it a notable contribution to the field of rectifier circuit design.

**Table 2:** Comparison between the proposed rectifier and existing works.

Frequency (GHz)	Input power	Size (mm)	Efficiency	Reference
1.8–2.6	−15dBm	110x110	30%	[27]
2.45	-5dBm	30 × 18	51%	[28]
2.025–2.36	-5dBm	70 × 66	50.65%	[29]
1.8–2.4	−10dBm	–	18 %	[30]
0.9-1.8-2.12-2.4	−10dBm	160x160	52%	[31]
0.850-1.81-2.18-2.4	−20dBm	160x160	48%	[32]
2.45	-5dBm	17x27	52%	This work

## 7 Conclusion

This paper has illuminated the entire process of designing, optimizing, and practically implementing an RF energy harvesting circuit. The outcomes of this study bring significant value to energy harvesting, with a clear emphasis on elevating energy efficiency and advocating for sustainable wireless power solutions. Through rigorous experimentation and detailed simulations, we have effectively showcased the feasibility and effectiveness of our RF energy harvesting circuit. The generated antenna has a gain of about 4 dBi and a bandwidth of 100 MHz while operating in the wifi band.

Our results clearly illustrate that our device achieves a remarkable peak efficiency of 52% under an input power of −5 dBm, coupled with a load resistance of 1600 Ω. These findings represent a substantial advancement in energy harvesting, positioning our work as a noteworthy contribution to the broader mission of harnessing and efficiently utilizing ambient RF energy resources.

## Data availability

No data were used for the research described in this paper.

## Funding

This work is funded and supported by the Deanship of Graduate Studies and Scientific Research, 10.13039/501100006261Taif University.

## CRediT authorship contribution statement

**Abdelmalek Reddaf:** Writing – review & editing, Writing – original draft, Visualization, Validation, Supervision, Software, Resources, Project administration, Methodology, Investigation, Funding acquisition, Formal analysis, Data curation, Conceptualization. **Mounir Boudjerda:** Writing – review & editing, Writing – original draft, Visualization, Validation, Supervision, Software, Resources, Project administration, Methodology, Investigation, Funding acquisition, Formal analysis, Data curation, Conceptualization. **Islem Bouchachi:** Writing – review & editing, Writing – original draft, Visualization, Validation, Supervision, Software, Resources, Project administration, Methodology, Investigation, Funding acquisition, Formal analysis, Data curation, Conceptualization. **Badreddine Babes:** Writing – review & editing, Writing – original draft, Visualization, Validation, Supervision, Software, Resources, Project administration, Methodology, Investigation, Funding acquisition, Formal analysis, Data curation, Conceptualization. **Ali Elrashidi:** Writing – review & editing, Writing – original draft, Visualization, Validation, Supervision, Software, Resources, Project administration, Methodology, Investigation, Funding acquisition, Formal analysis, Data curation, Conceptualization. **Kareem M. AboRas:** Writing – review & editing, Writing – original draft, Visualization, Validation, Supervision, Software, Resources, Project administration, Methodology, Investigation, Funding acquisition, Formal analysis, Data curation, Conceptualization. **Enas Ali:** Writing – review & editing, Writing – original draft, Visualization, Validation, Supervision, Software, Resources, Project administration, Methodology, Investigation, Funding acquisition, Formal analysis, Data curation, Conceptualization. **Sherif S.M. Ghoneim:** Writing – review & editing, Writing – original draft, Visualization, Validation, Supervision, Software, Resources, Project administration, Methodology, Investigation, Funding acquisition, Formal analysis, Data curation, Conceptualization. **Mahmoud Elsisi:** Writing – review & editing, Writing – original draft, Visualization, Validation, Supervision, Software, Resources, Project administration, Methodology, Investigation, Funding acquisition, Formal analysis, Data curation, Conceptualization.

## Declaration of competing interest

The authors declare that they have no known competing financial interests or personal relationships that could have appeared to influence the work reported in this paper.
